# Supplementary material for: Understanding target-specific effects of antidepressant drug pollution on molluscs: A systematic review protocol
Source: PLoS One. 2023 Jun 27;18(6):e0287582. doi: 10.1371/journal.pone.0287582 (PMC10298782; doi:10.1371/journal.pone.0287582)
Supplement: Attachment — Submitted filename: Response to Reviewers.docx [file pone.0287582.s003.docx]

Environmental Science Division,

Department of Life Sciences,

College of Health, Medicine and Life Sciences (CHMLS),

Brunel University London, Uxbridge, UB8 3PH, UK.

May 10, 2023

Professor Peter P. Fong

Academic Editor,

PLOS ONE

Dear Prof. Fong,

**A rebuttal letter for the submission (PONE-D-23-08500) : Understanding target-specific effects of antidepressant drug pollution on molluscs: a systematic review protocol**

I would like to thank you for the timely and comprehensive peer review of our manuscript and for your decision.

I must say we are particularly pleased and fortunate to have you as the editor of our manuscript considering the match, which is often uncommon.

We are also very indebted to the peer reviewers for their time, efforts and for providing invaluable comments for improving the overall quality of our manuscript.

All points raised during the review process are hereby addressed as follows:

| **Reviewers’ comments** | **Authors’ response** |
| --- | --- |
| **Academic editor**: |  |
| Pay close attention to and respond to the comments of all three reviewers, but especially of reviewers 1 and 3. In particular, reviewer #1 *says that in molluscs, the mode of action is more complicated than simply modulation of monoaminergic pathways and I agree with this*. *So, it would be appropriate to expand your search to include alternate modes of action of antidepressants in molluscs*. There are published papers on these alternative modes of action. | I would like to thank you for aligning, in particular, with this observation. However, the need to clearly include modes of action other than the monoaminergic pathways will not affect the search strategy but only the inclusion criteria as the search strings were developed to retrieve all articles on antidepressants and molluscs, including those on alternate modes of action.  Indeed, we do understand that target-specific effects of antidepressants on molluscs may not be defined ‘categorically’ by the monoaminergic system alone, and as a result, the term ‘neurophysiology’ was used to describe other processes that may be implicated, perhaps, this was not clear enough.  This has now been rephrased and the term intracellular signalling events (in addition to neurophysiology) has also been added to the inclusion criteria – this will describe other pathways (*and not necessarily systems*) that may be implicated in addition to the comprehensive list of physiological systems already provided that are regulated by the monoaminergic system in molluscs. |
| **Reviewer 1** |  |
| This represents a timely approach in the evaluation of the impact of pharmaceuticals in this invertebrate group.  *However, the criteria for inclusion should be better justified*. Although the aim of this work is to extract relevant literature data and not descriptive information, *the choice of these criteria is extremely narrow, based on the assumption that target-specific effects of antidepressants in molluscs are restricted to the monoaminergic system,* which is currently not taken for granted even in humans. | I would like to thank the reviewer in particular for this key observation. This observation on the inclusion criteria has now been rephrased and expanded as initially provided in the response above. |
| The ms. is clear and well written.  *Only subheadings are not* clear (i.e. Methods and subheadings of the following sections  are the same) and should be revised. | Thank you for the comment. However, evidence synthesis practice relies on discipline-specific institutional/community guidelines in the conduct and reporting of studies. In environmental sciences, these guidelines/reporting standards are provided by the Collaboration for environmental evidence (CEE), much the same way as Cochrane (formally Cochrane collaboration) for evidence synthesis in clinical medicine.  The manuscript (our protocol) outline (all sections and sub-sections) is written in line with the RepOrting standards for Systematic Evidence Syntheses (ROSES) by CEE (<https://www.roses-reporting.com/systematic-review-protocols>) |
| The statement …. physiological processes (including reproduction and development) are regulated by monoamines rather than vertebrate-type sex steroids in molluscs is obviously correct.  *However, in the introduction, a paragraph is lacking underlying the profound differences between the endocrine system in*  *humans as vertebrates with respect to molluscs, where chemical communication is mainly neuroendocrine, and endocrine*  *glands do not exis*t.  *For those not familiar with molluscan neuroendocrine system the examples made in paragraph following line 118 are not explanatory enough.* | Additional information on the ‘neuroendocrine system’ of molluscs for some elucidation, as may be necessary, has now been provided. |
| 134-135 ecologically unique body forms, sizes, lifestyles, and microhabitat preferences : *delete ‘ecological’* | Thank you for the observation.  The argument here, however, is that the great diversity in their morphology also reflects their wide range of unique adaptation to the environment – their ecology. The current form serves this purpose better. |
| 139 in the range commonly = *in the range of those commonly* | Thank you for the observation, *this has now been corrected.* |
| 139-141 it should be specified that this sentence refers to a 2010 OECD guideline for Mollusc Reproductive Toxicity Tests in freshwater gastropods | The reference here is an OECD **detailed review paper** (DRP) on *molluscs life-cycle toxicity testing* (***not*** *a* ***guideline*** *on Mollusc Reproductive Toxicity Tests in freshwater gastropods*).  The DRP explains need to understand effects (especially on reproduction and development) of chemicals (in addition to EDCs) with unique potency for non-arthropod invertebrates (molluscs in particular, not necessarily freshwater gastropods).  *The sentence in its current form (with the OECD DRP reference) does justice to this.* |
| We understand the reasons for exclusion criteria as in Table 2.  *However, the Authors should consider a wider point of view*. Although molluscs are supposed to express all the components of monoamine signaling, their occurrence has been demonstrated only in few species, which should be taken into account.  *Moreover, the outcome measures chosen as* ***inclusion criteria do not consider mechanisms of action of antidepressants other than interference*** ***with the monoaminergic system***.  However, it is still unclear how antidepressants work even in humans. I suggest to take into account, or at least mention, other mechanisms of action, in order to better clarify the focus of this review protocol.  See for example Kornhuber, J.; Gulbins, E. New Molecular Targets for Antidepressant Drugs. Pharmaceuticals 2021, 14, 894. https:// doi.org/10.3390/ph14090894 and refs. therein. | Thank you very much for the emphasis.  This is the same as the first two comments, and have now been expanded to include other possible modes of action. |
| 271 : Potential effect modifiers:  Sex, reproductive strategy, reproductive stage **are not considered**. This should be justified, since these biological variables can strongly influence the response to antidepressants in different molluscan species | *These variables - biological and those of the experimental conditions, are by no means exhaustive, and depending on the actual data/outcome, can allow for some flexibility in the discussion*.  Nonetheless, ‘sex’, ‘reproductive strategy’ and ‘life stage’ (AND NOT ‘reproductive stage’ with life stage as a more broad-based description) have now been included in the potential effect modifiers. |
| **Reviewer #2** |  |
| The manuscript by Imiuwa et al describes the methodology used by the authors to perform a systematic review of the literature on the effects of antidepressants on aquatic molluscs. If performing a systematic review on this topic could be of interest, allowing to highlight knowledge gaps (as the authors mention in their introduction), **I do not understand the aim/interest of the present manuscript**. ***Only the methodology of the review is described, no results, no discussion.***  *There is nothing special or any novelty about the methodology used, it is the standard methodology of* ***a meta-analysis***. | Thank you for the comments.  While it is generally acknowledged that evidence synthesis (e.g. Systematic review protocols, systematic reviews, systematic evidence map protocols, systematic evidence maps and synthesis methods) in environmental science is relatively new compared to its applications in clinical medicine, the peer-review validation of ‘systematic review protocols’ in environmental science – *for methodology* *rigor*, *transparency* and *reproducibility*, is increasingly becoming standard practice.  *A systematic review refers to a research synthesis that employs pre-established, consistent and transparent methods to identify and evaluate all available research and information relevant to a research question, or phenomenon.*  It uses high-quality systematically applied transparent procedures to search, select, evaluate and synthesize evidence relevant to a key research question. *Its goal is to use these rigorous and transparent procedures* ***to reduce*** *the influence of* ***reviewer bias*** *(in both study selection and evaluation) and* ***error*** *in the evaluation process.* Systematic reviews are critical to evidence syntheses using primary research data.  All procedures are explicitly defined in advance (via a systematic review protocol).  Please see published protocols:  <https://doi.org/10.1186/s13750-022-00255-1>  <https://doi.org/10.1002/2688-8319.12057>  There are now published guidelines for the conduct and reporting of both systematic review protocols and systematic reviews in environmental science (including how they should be reviewed for journal publication):  ROSES reporting standard (by CCE)-  <https://environmentalevidence.org/cee-centres>  COSTER-  <https://doi.org/10.1016/j.envint.2020.105926>  *A narrative or traditional review, on the other hand, refers to a review carried out without a predefined conduct and reporting framework, and content and presentation are generally based on ‘expert’ judgement. Its purpose is to locate a few studies that describe the problem of interest.* |
| *Moreover, it seems that at the end the systematic review will become a narrative review as the extracted data were not amenable. Thus, why not present this review directly?* | Using standard, valid, rigorous, transparent and systematically applied methods, the procedures for systematic reviews include:   1. Problem statement formulation 2. Protocol development, registration and peer review publication 3. Literature search 4. Literature selection 5. Risk of bias assessment 6. Data extraction 7. Data synthesis and discussion: a. Narrative analysis (synthesis), or b. Meta-analysis   So, the narrative analysis of data that is systematically generated from a collection of primary research does not make such *synthesis* a traditional or narrative review.  Meta-analysis, is subjecting data from a collection of primary research to further statistical analysis, and as a rule, such dataset must be largely homogenous for meta-analysis to be valid. Human clinical intervention data, and not animal ecotoxicological data, are largely amenable to meta-analysis. |
| As the difference between “narrative review” and “systematic review” may not be clear to every reader, add few words to  explain the difference. | This will be superfluous as there are guidelines (including preferred reporting items) for conducting and *reporting* of systematic reviews. Besides, environmental science literature is now replete with information on evidence synthesis practice. |
| If target-proteins are well defined in humans, as the author said this is not perfectly understood in molluscs. So, what are “target-specific effects” and their links to the selected outcomes? | *What* ***are not*** *clearly understood* in molluscs are the effects (of low or environmental levels of different classes of antidepressants) on the drug-targets, *not the targets.* |
| Some explanation come l.231-235, but it may be a bit late. | This is a methodology manuscript (based on reporting guidelines) - the explanation here is placed under the appropriate section based on these guidelines. |
| Explain the chosen keywords.  Why do you target specific compounds and not stay at the family level using SSRIs, TCAs, SNRIs, etc. (as described in the introduction)? Or both specific compounds and families? No active metabolites were chosen as keywords while they are mentioned in the PECO question, why? | The family level description will not have chemical identity, and organisms are exposed to specific antidepressant. All active metabolites (or all the parent compounds) of over 10 different antidepressants currently in used cannot appear as key words. |
| Table 1. Remove capital letters and use only lower-case letters. | This is the correct search string syntax on the databases. |
| As metabolites are also compounds of interest, it is not clear why studies on biotransformation were excluded. CYP450 proteins can also be defined as a target of drugs. | The specific targets here are those for therapeutic drug actions, and not the ‘general’ proteins that cleave drug molecules in order to biotransform them for clearance. |
| L281. Why did the author consider the data as not amenable? | The comment here has been responded to above. |
| **Reviewer #3** |  |
| The review proposed on antidepressant drug pollution effects on molluscs meets the interest of scientists involved in the field of emerging contaminants in the aquatic environment. They may be lab researchers, depuration plant engineers, as well as regulators focused on prioritization to counteract environmental pollution.  Evidence mainly dating back in 2014-15 suggested that molluscs are much more vulnerable to the effects of antidepressants with respect to animals from other phyla. In the latest years, studies and results were more fragmented since addressed to many parameters and obtained in different animal models, thus a systematic review is advisable to reach a wide and updated vision | No issue raised.  Thank you for the evaluation. |
| I agree with the point of view presented by the authors on the absence or different function of enzymes in molluscs versus vertebrates (the example at line 120-123 and later on), and I support their willing to go in-depth on this point. Too many papers claim for different function alterations while referring only to different gene expressions, but it is not always the case. | No issue raised |
| Also, I agree with the search on the three databases reported in Table 1.  *At page 11, lines 225-226 active metabolites of antidepressants are mentioned, but they are not in the list of Table 1* | Thank you for the comment.  As active metabolites of antidepressants are named using derivation chemical reaction prefixes ( e.g. N-desmethyl**venlafaxine**, O-desmethyl**venlafaxine**, for Venlafaxine), with the name of the parent compounds still part of the names of their metabolites; the search string will retrieve any article using the name of the parent compounds.  Additionally, in both search strings, the term ‘antidepressant’ in addition to ‘psychotropic drugs’ is used, this is designed to be able to retrieve all articles where the word ‘antidepressant’ is used on either the title, abstract, key words or the general text, regardless of the specific antidepressants or metabolites. |
| As to the outcome, although the in vitro studies are excluded from the review, *I would extend the article screening also to “cell physiology*” *not only neurophysiology* since many effects regard cell receptor interaction, modulation of cell also signaling, organelles function, etc.  In bivalve early life stages in vivo studies are available on antidepressants that are not addressed strictly to as development | Thank you for the comment.  Reviewer #1 made the same observation, and the inclusion criteria has now been expanded to include ‘intracellular signaling events’ in addition to ‘neurophysiology’. |
| How will the authors identify and select the “grey” literature?  *This is not mentioned*, *however I suggest doing it only from official Reports and from Thesis that are hopefully validated by supervisors at specific Universities following the same criteria proposed in Table 2 and 3.* | Thank you for the comment.  However, *the subsection* ‘**Searching for articles**’ has information on a comprehensive search for grey literature:  *“Further, as supplementary searches*  *for grey literature in catalogues of academic theses, databases of conferences and proceedings, preprint servers and funders’ databases of on-going research have been recommended for mitigating publication bias in systematic reviews, additional searches will be conducted in ProQuest Dissertations and Theses Global, Open Access Theses and Dissertations, OpenGrey, Grey Literature Report, Research square and EcoEvoRxiv for grey literature. The supplementary searches will be carried out using key study terms”*  Accordingly, as the use of black literature search strings are not currently applicable in grey literature archives (or databases), only key words are used for search and retrieval (Adams J, Hillier-Brown FC, Moore HJ, Lake AA, Araujo-Soares V, White M, et al. : <http://dx.doi.org/10.1186/s13643-016-0337-y>). |

Additionally, I would like to bring to your notice that article number ‘44’ on the reference list of the original version of the manuscript ( being a response to comments to the authors) has now been replaced by the original article ( number ‘41’ on the revised manuscript, and previously cited as 41 before revision). This change could not be highlighted as tacked changes as it was effected using a reference manager.

I am happy to provide additional information or clarification as may be necessary.

Thank you very much for your time and the opportunity to submit a revised manuscript.

Yours sincerely,

Eghosa M. Imiuwa (on behalf of the authors)

Doctoral researcher

Division of Environmental Sciences,

Brunel University London, UK
